# Supplementary material for: Content-rich biological network constructed by mining PubMed abstracts
Source: BMC Bioinformatics. 2004 Oct 8;5:147. doi: 10.1186/1471-2105-5-147 (PMC528731; doi:10.1186/1471-2105-5-147)
Supplement: Additional File 2 — The original results of the above study (non-essential files are deleted to keep the file size under the limit set by BMC bioinformatics). [file 1471-2105-5-147-S2.bz2 › chilibotAdditionalFile2/dip40/706ID10734142E18043/html/MUTS_UVRD.html]

 


 **MUTS** and **UVRD** 
  
Found 31 abstracts in PubMed, retrieved 31.  
 

 What does Google say? 
 PDF only 
| .edu only 

---

**Interactive relationship** (e.g. stimulation, inhibition, etc)

**Inhibitory relationship**- This phenotype is suppressed by a  **uvrD**  mutU defect, and partially suppressed by mutH, mutL, or  **mutS**  mutations.  Ref: 11381137 Proc Natl Acad Sci U S A, 2001
**Neutral relationship**- **MutS**  and MutL activate DNA helicase II  [ **UVRD** ]  in a mismatch dependent manner.  Ref: 9535910 J Biol Chem, 1998
- All possible pairwise combinations of  **UvrD** , MutL,  **MutS** , and MutH were tested using the yeast two hybrid system to identify potential interactions involving mismatch repair proteins.  Ref: 9880500 J Biol Chem, 1999
- Hyper recombination was found for  **mutS** , mutL, mutH = mutR and mutU =  **uvrD** , with the largest effects seen for certain alleles of  **uvrD** .  Ref: 3519362 Genetics, 1986

**Non-interactive relationship** (e.g. studied together, co-existance, homology, etc.)

- Mapping and genetic characterization indicate that each tex gene corresponds to a previously identified gene involved in cellular DNA metabolism recB and or recC,  **uvrD** , mutH,  **mutS** , and dam.  Ref: 6287993 Basic Life Sci, 1982
- Escherichia coli  **MutS** , MutL, and DNA helicase II  [ **UVRD** ]  are sufficient to initiate mismatch dependent unwinding of an incised heteroduplex Yamaguchi, M.  Ref: 9535911 J Biol Chem, 1998
- We screened the following host mutator mutations for effects on spontaneous mutation rates in T4 mutT destruction of aberrant dGTPs, polA, polB and polC DNA polymerases, dnaQ exonucleolytic proofreading, mutH,  **mutS** , mutL and  **uvrD**  methyl directed DNA mismatch repair, mutM and mutY excision repair of oxygen damaged DNA, mutA function unknown, and topB and osmZ affecting DNA topology.  Ref: 7851754 Genetics, 1994
- We also describe the alterations in the  **mutS** , mutL and  **uvrD**  genes responsible for the mutator phenotype of hypermutable P. aeruginosa strains isolated from CF patients.  Ref: 11952911 Mol Microbiol, 2002
- We present novel phylogenetic evidence for horizontal transfer of three genes involved in DNA replication and repair  **mutS** ,  **uvrD** , and polA.  Ref: 11746762 Environ Mol Mutagen, 2001
- **MutS** , MutL, and DNA helicase II  [ **UVRD** ]  are required for the mismatch provoked excision step that occurs during Escherichia coli methyl directed mismatch repair.  Ref: 9535910 J Biol Chem, 1998
- We show that 21 additional tex mutations with a mutator phenotype map to five genes previously identified as components of a methylation directed pathway for repair of base pair mismatches  **uvrD** , mutH, mutL,  **mutS**  and dam.  Ref: 2981756 Genetics, 1985
- **MutS**  and MutL dependent activation of DNA helicase II  [ **UVRD** ]  has also been demonstrated with a model substrate in which a 138 residue oligonucleotide was hybridized to a 138 nucleotide gap in an otherwise duplex 00 base pair circular DNA.  Ref: 9535910 J Biol Chem, 1998
- However, this correction reaction did not require mutH, mutL,  **mutS** , or  **uvrD**  gene products.  Ref: 3550791 Proc Natl Acad Sci U S A, 1987
- Co repair of symmetrically methylated heteroduplex DNA required the  **mutS**  and  **uvrD**  gene products, while repair of unmethylated heteroduplex DNA also required the mutL and mutH gene products.  Ref: 3522919 J Mol Biol, 1986
- Unwinding of both substrates in the presence of  **MutS** , MutL, DNA helicase II  [ **UVRD** ] , and single stranded DNA binding protein was mismatch dependent and initiated at the single strand break.  Ref: 9535911 J Biol Chem, 1998
- Mutagenesis, by methylating and ethylating agents, in mutH, mutL,  **mutS** , and  **uvrD**  mutants of Salmonella typhimurium LT2.  Ref: 6401281 J Bacteriol, 1983
- To investigate the effects on PV rates of mutations in genes for mismatch repair MMR, insertion deletion mutations of  **mutS** , mutL, mutH, dam, polI,  **uvrD** , mfd and recA were constructed in Hi strain Rd.  Ref: 11889052 EMBO J, 2002
- The increased susceptibility to mutagenesis by small alkylating agents due to mutH, mutL,  **mutS** , and  **uvrD**  mutations was not accompanied by an increased sensitivity to killing by these agents.  Ref: 6401281 J Bacteriol, 1983
- The Escherichia coli methyl directed reaction has been reconstituted in a purified system consisting of MutH, MutL, and  **MutS**  proteins, DNA helicase II  [ **UVRD** ] , single strand DNA binding protein, DNA polymerase III holoenzyme, exonuclease I, DNA ligase, along with ATP adenosine triphosphate, and the four deoxynucleoside triphosphates.  Ref: 2665076 Science, 1989
- Two mutators mapped at the position of the mutH and mutL loci of S. typhimurium, and two other mutators mapped at positions corresponding to the  **mutS**  and  **uvrD**  loci of Escherichia coli.  Ref: 6268610 J Bacteriol, 1981
- The increases in spontaneous reversion of histidine mutations were first measured in isogenic strains carrying mismatch repair defective mutH, mutL,  **mutS** , or  **uvrD**  alleles, relative to their mismatch repair proficient counterparts.  Ref: 9685594 Mutat Res, 1998
- The increased frequency of base substitution mutations was recA independent in the mutH, mutL, and  **uvrD**  strains and partially recA independent in the  **mutS**  strain.  Ref: 6268610 J Bacteriol, 1981
- Deletion rates were enhanced 30 to 50 fold by the mismatch repair defects,  **mutS** , mutL and  **uvrD** , and 250 fold by the ssb 3 allele, but the effect of the mismatch defects was canceled by the delta recA allele.  Ref: 9055067 Genetics, 1997
- As judged by protein affinity chromatography, MutL and MutL E32K both support formation of ternary complexes that also contain  **MutS**  and MutH or  **MutS**  and DNA helicase II  [ **UVRD** ] .  Ref: 10734142 J Biol Chem, 2000
- The in vitro activity was also dependent on products of mutH, mutL,  **mutS** , and  **uvrD**  loci and was equally efficient on nucleotide insertions and deletions.  Ref: 9278429 J Biol Chem, 1997
- Seven out of the 11 mutator strains were found to be defective in the MMR system four  **mutS** , two mutL and one  **uvrD** .  Ref: 11952911 Mol Microbiol, 2002
- By genetic analysis, we show that the inhibitory effect of ssDNA exonucleases on deletion formation is independent of the  **MutS**  and  **UvrD**  proteins.  Ref: 12538867 Proc Natl Acad Sci U S A, 2003
- Repair of both heteroduplex orientations requires MutH, MutL,  **MutS** , DNA helicase II  [ **UVRD** ] , SSB, and DNA polymerase III holoenzyme, but the two substrates differ with respect to exonuclease requirements for correction.  Ref: 8389365 J Biol Chem, 1993
- We have used artificially constructed heteroallelic heteroduplex molecules of bacteriophage lambda DNA to transfect Escherichia coli, and E. coli mutants deficient in various functions involved in the adenine methylation directed mismatch repair system, MutL,  **MutS** , MutH, and  **UvrD**  MutU.  Ref: 2961649 Genetics, 1987
- The results shown here indicate that the putative P. aeruginosa  **mutS** , mutL and  **uvrD**  genes are mutator genes.  Ref: 11952911 Mol Microbiol, 2002
- Salmonella typhimurium LT2 mutH, mutL,  **mutS** , and  **uvrD**  mutants were especially sensitive to mutagenesis by both the recA dependent mutagen methyl methane sulfonate and the recA independent mutagen ethyl methane sulfonate, but not to mutagenesis by agents such as 4 nitroquinoline 1 oxide and UV irradiation.  Ref: 6401281 J Bacteriol, 1983
- Displacement of the oligonucleotide requires  **MutS** , MutL, DNA helicase II  [ **UVRD** ] , and ATP and is dependent on the presence of a mismatch within the hybrid region.  Ref: 9535910 J Biol Chem, 1998
- In Test A, repair proficient strain and its uvrA counterpart are used for detection of spontaneous and UV induced mutations, while in Test B mismatch repair deficient strains mutH,  **mutS** , mutL and  **uvrD**  are used for amplified detection of spontaneous mutations caused by replication errors.  Ref: 9675243 Mutat Res, 1998
- The mismatch repair system  **mutS** , mutL and  **uvrD**  genes in Pseudomonas aeruginosa molecular characterization of naturally occurring mutants.  Ref: 11952911 Mol Microbiol, 2002
- The mode of mismatch conversion observed in these experiments was independent of the host mutH, mutL,  **mutS** , and  **uvrD**  genes.  Ref: 3536859 J Bacteriol, 1986
- The growth defect and filamentation phenotypes associated with 2 aminopurine exposure are effectively suppressed by null mutations in mutH, mutL,  **mutS** , or  **uvrD**  mutU, which encode activities that act upstream of the four exonucleases in the mechanism for the methyl directed reaction that has been proposed based on in vitro studies.  Ref: 11381137 Proc Natl Acad Sci U S A, 2001
- The most efficient co repair pathway was the Dam instructed repair pathway that required the mutH, mutL,  **mutS**  and  **uvrD**  gene products and preferentially used the methylated strand as the template for DNA synthesis.  Ref: 3522919 J Mol Biol, 1986
- This system involves the functions of mutH, mutL,  **mutS**  and mutU  **uvrD**  loci which recognize mispaired bases as a consequence of errors due to the polymerase itself.  Ref: 3537315 J Mol Biol, 1986
